# Contribution of Emotion Dynamics to Adolescent Psychosocial Well-Being: Protocol for a Longitudinal Study

**DOI:** 10.2196/76333

**Published:** 2026-02-18

**Authors:** Carli Mastronardi, Jade Powers, Rosanne Menna, Lance M Rappaport

**Affiliations:** 1 Department of Psychology University of Windsor Windsor, ON Canada

**Keywords:** emotion dynamics, daily diary, psychosocial well-being, adolescence, academic motivation, internalizing syndromes

## Abstract

**Background:**

As a critical period in psychosocial development, adolescence is marked by heightened emotion regulation demands as well as increased risk for, and vulnerability to, stress.

**Objective:**

This longitudinal study investigates how dynamic patterns (ie, mean intensity, variability, instability, inertia, and reactivity to stress) in positive and negative affect relate to, and predict change in, broad domains of adolescent psychosocial well-being (ie, mental health, social well-being, and academic motivation). Using a daily diary procedure to capture adolescents’ daily naturalistic affective experiences, this study will provide novel insights into how affective processes predict psychosocial well-being over time beyond traditional, static assessment.

**Methods:**

At baseline, adolescents aged 14-17 years from Southwestern Ontario reported on their academic motivation (eg, extrinsic motivation), social well-being (eg, social support and loneliness), and mental health (eg, anxiety syndrome severity) before completing a 35-day smartphone-based daily diary protocol wherein participants reported twice daily in the morning (ie, 7-10 AM) and evening (ie, 8-11 PM) on positive and negative affect, stress, and internalizing symptom severity. Participants then repeat the surveys of academic motivation, social well-being, and mental health 6, 12, and 18 months following baseline assessment to assess change in each domain of psychosocial well-being over time.

**Results:**

Adolescents (N=149) were enrolled into this longitudinal study between April 2023 and November 2024, such that all participants will complete the scheduled 18 months of longitudinal follow-up assessments by May 2026. Primary study analyses will use multilevel modeling, structural equation modeling, multilevel structural equation modeling, and dynamic structural equation modeling to examine how dynamic patterns in positive and negative affect (eg, instability, inertia) concurrently correlate with, and prospectively predict change in, psychopathology and well-being.

**Conclusions:**

This study protocol paper outlines the overarching study objectives and methodology to promote transparency and reproducibility. Through the integration of daily diary methodology within a longitudinal design, this study aims to clarify the potential implications of dynamic affective processing (eg, affective reactivity to daily stress) for both adolescent psychopathology and well-being beyond clinical syndromes.

**International Registered Report Identifier (IRRID):**

DERR1-10.2196/76333

## Introduction

### Background

Adolescence presents a critical period for psychosocial and socioemotional development including the development and emergence of many internalizing syndromes [1,2]. For example, epidemiological estimates suggest that 18%-22% of adolescents recently experienced psychopathology or related psychosocial distress [3-5], and psychiatric conditions often emerge during adolescence and then persist into adulthood [6,7]. Adolescent psychosocial distress also impairs multiple areas of functioning including social interactions, academic performance [8], and overall well-being [9]. While longstanding theory documents acute affective distress in adolescence [10], psychosocial distress and related psychopathology may have become particularly prevalent and severe in recent years. For example, the prevalence of internalizing psychopathology rose markedly from 2007 to 2017 [11] and from 1990 to 2023 [12,13]. Similarly, youth visits to emergency departments for mental health–related distress increased substantially from 2009 to 2015 [14].

Historically, research on child and adolescent mental health focused on stable factors that predict psychopathology, such as personality traits [15], temperament [16], and attachment styles [17]. However, despite their theoretical stability and strong test-retest reliability [18,19], each of these seminal theories also encompasses constructs of variability or instability, such as impulsiveness [20], negative affectivity [21,22], or reactivity [23], that confer risk to develop psychopathology. These theories of psychopathology risk suggest that both stable traits and dynamic fluctuations, such as change in response to the demands of one’s environment, may provide unique and important insights into adult well-being and adolescent psychosocial and socioemotional development [24-26]. For example, emotional reactivity to stressful events is associated with psychopathology (eg, depression and anxiety) in adolescence [27] and early adulthood [28]. Similarly, depressive symptoms in adults have been implicated in elevated mean quarrelsome and submissive behavior [29], as well as reduced responsiveness of interpersonal behavior to affective [30] and situational cues [31] that typically prompt one to adapt their behavior to the situation [32,33]. However, while considerable extant research examined stable correlates of psychopathology risk in adults and adolescents, there is a paucity of research to examine naturalistic dynamics in adolescence that reflect how adolescents adapt to environmental demands, such as daily stressful experiences. Hence, further research is needed to explore the potential implications of naturalistic dynamics (eg, in emotions) to offer important insights into adolescent psychosocial and socioemotional development above and beyond traditional static, stable indictors of risk.

Extant research on in situ emotional experiences suggests that affective states are inherently dynamic and fluctuate over time (see Kuppens et al [34] for review). Many theories of emotion emphasize the role of acute fluctuations in momentary positive and negative affect to help one (1) locate suitable environments, (2) attend to changes in the environment, (3) appraise the situation, and (4) choose effective compensatory, regulatory behaviors with the goal of returning to emotional equilibrium (see Frijda [35], Gross and Thompson [36], and Scherer [37] for review). Dynamics in one’s affective experiences over time may summarize data on a set of momentary affective experiences, such as affective instability or variability over time, to index affective difficulties or dysregulation [38,39]. For example, affective variability over time and instability from one occasion to the next may predict various clinical outcomes (eg, depressive symptoms) and related indicators of psychosocial well-being [24,26].

However, most research on the clinical implications of in situ affective fluctuations and related emotion dynamics focused on adulthood [24]. Less research examined the potential implications of in situ affective dynamics in adolescence [40]. The available evidence suggests that, relative to adults, adolescents may report more intense and variable affective fluctuations [41,42] consistent with heightened challenges during adolescence [10,43]. For example, relative to adults, adolescents reported greater positive and negative affective instability and heightened affective intensity, which may decrease over time as they reach adulthood [41,44]. Given that adolescence may pose a novel challenge for youth to manage more intense, variable, and dynamic emotions [10,41,45], research is critically needed to understand the manifestation of affective dynamics in adolescence and its implications for adolescent well-being including both psychopathology and psychosocial development.

Technological and statistical innovations (eg, the proliferation of smartphones) facilitate the repeated assessment of affect as it manifests naturalistically during adolescents’ daily lives. Referred to as experience sampling methodologies, including daily diary procedures, this class of methodology facilitates assessment of affect on the temporal scale required to accurately test many psychological theories about well-being (see Shiffman et al [46] for review). For example, per the self-medication theory, individuals may engage in high-risk alcohol use to reduce momentary affective distress [47]. However, past research largely relied on cross-sectional or longitudinal evidence of high-risk alcohol use among people *who* experience distress (eg, anxiety disorders; see Turner et al [48] for review). In contrast, experience sampling methodology research identified that acute, severe negative affect may predict *when* at-risk young adults engage in high-risk alcohol use [49,50]. Experience sampling methods, such as daily diary procedures, also naturalistically assess participants’ current or recent affect, stressful experiences, and social context, which minimize recall biases typically associated with self-report (eg, by minimizing participants’ recall time) and provide greater ecological validity than laboratory-based tasks (eg, through the assessment of events in situ) [51,52].

### Emotion Dynamics

Emotion dynamics characterize the trajectories, patterns, and regularities of how positive and negative affect vary over time [24,53]. Experience sampling and daily diary methods [54] previously identified 4 dynamics that have been the focus of most extant research: mean affective intensity, variability, instability, and inertia [26]. Mean intensity of positive and negative affect refers to the average level of positive and negative affect that a participant reported over a set of records completed over a specified period of time (eg, 30 days). Specifically, mean intensity is typically computed separately for positive and negative affect as the mean of all assessments of positive or negative affect provided. Among adults, elevated mean negative affect is consistently associated with psychopathology [24] including depressive and anxiety disorders [55,56]. During adolescence, mean positive and negative affect may be higher [44] and correlated with psychopathology [57,58].

Affective variability and instability capture intraindividual (ie, within-person) fluctuations in positive and negative affect around one’s mean level, such as variation over moments or days. Affective variability and instability are typically computed separately for positive and negative affect. Specifically, affective variability is computed as the intraindividual standard deviation while affective instability is computed as the mean squared successive difference (MSSD) between consecutive assessments adjusted for the average time between consecutive assessments [59]. Hence, high variability in negative affect suggests that one’s affective experience varies greatly over the occasions studied whereas low affective variability suggests that one’s affective experience was consistently close to one’s mean level. Similarly, high instability in negative affect suggests frequent large changes in negative affect from one occasion to the next whereas low instability in negative affect would suggest that one’s negative affect is relatively static from one occasion to the next. Empirically in adults, instability and variability have been consistently, albeit modestly, associated with lower well-being and psychopathology [24,26,60]. Extensive prior research also consistently implicates affective variability and instability in adolescent psychopathology [61-63]. Given evidence of elevated affective variability and instability in adolescence [10,64], further longitudinal research is needed to examine whether affective variability or instability is associated with change in psychopathology over time. Meanwhile, further research is needed in adolescence to understand the implications of affective variability and instability for other facets of well-being during adolescent psychosocial development (eg, academic motivation).

Like the MSSD, inertia in positive or negative affect reflects the degree to which positive or negative affect at the last assessment (eg, moment or day) persists to influence one’s current positive or negative affect [65]. However, whereas the MSSD reflects deviations from the autocorrelation of positive and negative affect over time as affective instability, affective inertia directly assesses the autocorrelation as persistence of positive or negative affect over time. Hence, while conceptually similar, extant research distinguishes between MSSD and inertia, which are moderately correlated [24,66]. Specifically, one can regress each assessment of positive or negative affect on the immediately preceding assessment through multilevel, mixed-effects regression [65] or dynamic structural equation modeling (DSEM) and residual dynamic structural equation modeling [67-69], which has several benefits including latent variable centering [70] and the ability to adjust for possibly unique responding early in a daily diary procedure [71]. Conceptually, elevated inertia in negative affect may indicate difficulty downregulating (ie, reducing) negative affect, which could reflect persistent negative affect that contributes to, or results from, frequent or particularly severe distress [72]. Similarly, although somewhat less emphasized in extant research, reduced inertia in positive affect could reflect difficulty maintaining positive affect. Prior research documented an association of elevated inertia in negative affect with depressive disorders in adulthood [73,74] (see Thompson et al [75] for review), adolescence [76], and childhood [77]. Some extant research examined implications of inertia in positive or negative affect for other psychopathological syndromes among adults (see Dejonckheere et al [24] for review). However, little prior research examined the manifestations of inertia in positive or negative affect during the significant emotional development represented by adolescence or the implications of inertia in positive or negative affect for other internalizing syndromes (eg, anxiety disorders) and facets of psychosocial well-being during adolescence. Moreover, like research on affective variability and instability, longitudinal research is needed to examine whether inertia in positive or negative affect predicts change in psychopathology or other indices of well-being over time.

Beyond the 4 emotion dynamics described thus far, substantial extant research emphasizes the implications of affective reactivity to stress for adult and adolescent psychopathology and well-being. Broadly, reactivity to stress refers to the association of a stressor with an affective, behavioral, or physiological change. For example, using multilevel mixed-effects regression, DSEM, or residual dynamic structural equation modeling, one can regress current positive or negative affect on positive or negative affect at the preceding assessment to assess change over the given time interval as well as regress positive or negative affect on the degree of stress experienced during said interval to assess the association of stress with concurrent change in positive or negative affect. Moreover, the association of stress with positive or negative affect can be examined as both fixed and random effects to evaluate the average impact of stress on positive or negative affect as well as interindividual (ie, between-person) heterogeneity in, and moderation of, the strength of one’s reactivity of positive or negative affect to stressful experiences. Extant research on the association of affective reactivity to stress with psychopathology is mixed. Laboratory-based research largely suggests that depressive symptomatology is associated with blunted reactivity to stress (see Bylsma et al [78] for review) whereas naturalistic research (eg, daily diary and ecological momentary assessment methods) suggests that depressive symptomology in adulthood [73,79] and adolescence [80,81] may be associated with elevated affective reactivity to stress. While future research is needed to clarify this apparent discrepancy, additional research is also needed to evaluate whether affective reactivity to stress is specifically associated with depressive disorders relative to other internalizing syndromes [82], particularly in adolescence [83]. Further research is also needed to evaluate the potential implications of affective reactivity to stress prospectively on the development of internalizing psychopathology and psychosocial distress during the high-risk period posed by adolescence [11,84].

### This Study

As a period of significant socioemotional development, adolescence is marked by heightened vulnerability to psychosocial distress (eg, internalizing syndromes) and increased affective instability [42,64]. Extant research delineates characteristics of fluctuations in naturalistic affective experiences (ie, mean intensity, variability, instability, inertia, and reactivity to stress) that may correlate with well-being and psychopathology in adults and youth [26,39,45]. However, extant research focused on the implications of putative emotion dynamics (eg, inertia) for psychopathology in adults (see Dejonckheere et al [24] for review). Given the substantial affective development documented during adolescence [10], further research is needed to examine the manifestation and implications of putative emotion dynamics on psychopathology during adolescence [45]. Additionally, although most prior research focused on 5 core emotion dynamics (ie, mean intensity, variability, instability, inertia, and reactivity to stress), future research can extend beyond these indices to examine additional dynamics (eg, emotion-network density [24]).

Moreover, given the substantial psychosocial development documented during adolescence [85], further research is needed to examine the implications of emotion dynamics for adolescent well-being above and beyond psychopathology. Specifically, this study will examine how 5 putative emotion dynamics (ie, mean affective intensity, variability, instability, inertia, and reactivity to stress) correlate with and predict change in adolescent well-being (ie, social well-being, academic motivation, and mental health) over 18 months. Specifically, this study will evaluate hypotheses that elevated mean negative affect, lower mean positive affect, elevated variability and instability in positive and negative affect, elevated inertia in negative affect, lower inertia in positive affect, and elevated reactivity of positive and negative affect to stress will contemporaneously correlate with elevated adolescent psychopathology and prospectively predict increased adolescent psychopathology (eg, depressive and anxiety syndrome severity) over 18 months. For example, this study will evaluate the contemporaneous and prospective association of baseline affective variability, instability [26,31], and reactivity of positive or negative affect to stress [78,83] with elevated psychopathology in adolescence. Similarly, this study will examine whether inertia in positive or negative affect is uniquely associated with elevated depressive symptomatology relative to other psychopathological syndromes (eg, anxiety and eating-related symptomatology [65,72]).

Finally, this study will evaluate hypotheses that elevated mean negative affect, lower mean positive affect, elevated variability and instability in positive and negative affect, elevated inertia in negative affect, lower inertia in positive affect, and elevated reactivity of positive and negative affect to stress will contemporaneously correlate with worsened psychosocial well-being and prospectively predict worsened adolescent psychosocial well-being (eg, academic motivation and parent and peer relationship quality) above and beyond joint associations with psychopathology. For example, this study will evaluate hypotheses that each emotion dynamic (eg, elevated mean negative affect intensity and lower mean positive affect intensity) is correlated with and prospectively predicts worsened social well-being (eg, increased loneliness) and academic motivation (eg, increased amotivation) above and beyond joint associations with psychopathology (eg, depressive symptomatology).

This protocol paper serves to provide a comprehensive record to increase transparency and persistent documentation of the study methodology. For example, the resulting dataset may support additional, secondary projects (eg, on the intraindividual dynamics of change in psychopathology and social relationships). In these cases, this paper serves as a comprehensive account of the study purpose and of all study measures and procedures. Similarly, this protocol paper serves to clearly articulate and register a comprehensive overview of the study aims, background, and methods prior to the completion of data collection. Finally, this protocol paper provides an example framework for longitudinal research to examine the naturalistic manifestation and implications of socioemotional correlates and predictors (eg, emotion dynamics) of adolescent psychopathology and adolescent well-being above and beyond concurrent psychopathology.

## Methods

### Project Overview

This study uses a longitudinal design to examine the concurrent and prospective association of 5 putative emotion dynamics (ie, affective mean intensity, variability, instability, inertia, and reactivity to stress) with psychosocial well-being in adolescence. Specifically, this study explores how each dynamic correlates with and predicts change in psychopathology (eg, depressive symptomatology) as well as life satisfaction, social well-being, and academic motivation above and beyond psychopathology. Using daily diary assessment, this study assesses the naturalistic manifestation of positive and negative affect, stress, and distress during adolescence to clarify the in situ fluctuation of affect during adolescents’ daily lives and the potential implications of dynamic affective fluctuations for adolescents’ development.

### Recruitment

Adolescent participants aged 14-17 years were recruited from the Windsor-Essex region of Southwestern Ontario from April through May 2023, September through December 2023, February through May 2024, and September through November 2024. Recruitment information was shared with adolescents in the local community through multiple channels to increase the opportunity for adolescents to participate and, thereby, increase the representativeness and generalizability of the resulting sample. Specifically, recruitment information was shared via advertisements on prominent social media (eg, Facebook and Instagram), flyers posted in local community centers, and information provided to students and families by both local school boards. For example, recruitment flyers were (1) posted in schools, (2) sent out to families via email or online message, and (3) shared on school social media accounts. Participants were eligible for the study if they were aged 14-17 years and had access to a device running the iOS or Android OS operating system. There were no explicit exclusion criteria to maximize sample heterogeneity and generalizability by allowing a broad range of adolescents to participate.

This study initially planned to recruit 200 participants to provide 80% statistical power to detect moderate correlations at baseline using a cautious testwise a value of .005 (*r*=0.253) [86]. According to a Monte Carlo simulation of a latent change score model, which fixed a value at .05 [87] and accounted for 20% attrition, the intended sample would have also provided 80% statistical power to detect a moderate prospective association (β=.33) of each emotion dynamic (eg, inertia) with change in each well-being domain over each 6-month interval. However, participant enrolment was ended in November 2024 so that all participants could complete the scheduled 18-month follow-up assessment before the end of the project funding in May 2026.

### Ethical Considerations

Study procedures were cleared by the University of Windsor Research Ethics Board (number 21-098) on November 8, 2022. During an initial, in-person baseline session, a parent or legal guardian provided informed consent for each adolescent to participate in the study. Each adolescent participant then provided separate informed consent prior to participation in the study. Participants will receive financial compensation up to $140 for time spent participating in each component of the study. Specifically, based on the local minimum wage, each participant received $23.50 for up to 90 minutes spent completing the in-person baseline assessment, received up to $70 based on the number of daily diary surveys completed for time spent completing daily diary surveys, and will receive $15.50 per follow-up survey completed for up to 60 minutes completing each follow-up assessment. Where applicable, each participant also received $10 at the baseline assessment to offset the cost of parking at the university. Data are stored securely through assignment of an arbitrary, person-specific alphanumeric code that facilitates linkage of participant data across study components. Following the end of data collection, data will be deidentified to support secondary use of study data.

### Design and Procedure

**Figure 1:**
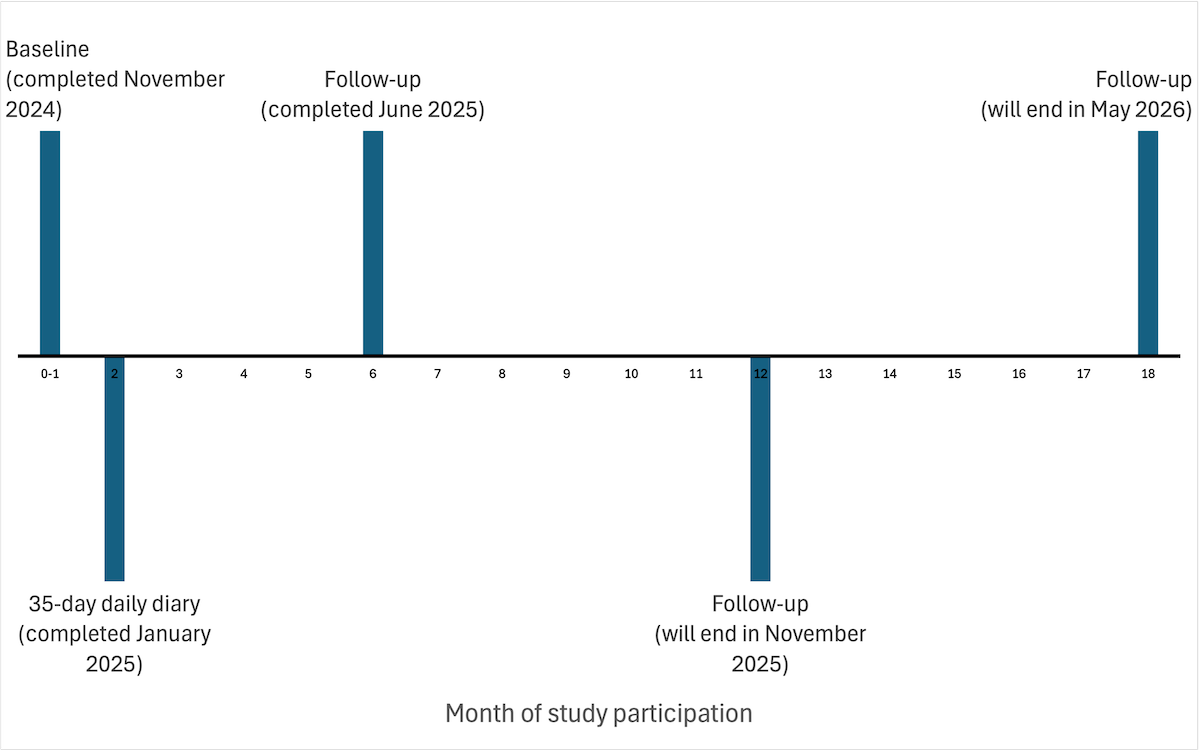
Study timeline and schedule.

This study uses a longitudinal design to examine concurrent correlates of, and prospective predictors of change in, adolescents’ psychosocial well-being over 18 months (Figure 1). Specifically, each participant was or will be asked to complete measures of social well-being (eg, loneliness and social support), academic motivation, and psychopathology at baseline as well as 6, 12, and 18 months following baseline assessment. For the 35 days immediately following baseline assessment, each participant was also asked to complete a 35-day daily dairy procedure to assess naturalistic dynamics in positive and negative affect (eg, inertia in positive affect and reactivity of negative affect to daily stress).

Specifically, following the provision of informed consent, each adolescent participant completed a baseline assessment, during which they completed online questionnaires that assess baseline social well-being, academic motivation, and mental health, and received instruction in the following daily diary procedure. A subset of participants also completed the Mini International Neuropsychiatric Interview for Children and Adolescents [MINI-KID], a structured interview used to assess risk for common psychopathological syndromes (see “Measures” section). Following baseline assessment, all participants completed a daily diary procedure for the next 35 days to assess naturalistic emotion dynamics, such as mean intensity of, and variability in, positive and negative affect. During this period, participants reported twice daily (ie, 7-10 AM and 8-11 PM) on positive and negative affect, anxiety and depressive symptoms, and stress experienced since waking. Thus, the timing of each assessment in the morning and evening assesses positive and negative affect, anxiety and depressive symptoms, and stress experienced immediately upon waking and over the course of each day, respectively. Three hours were provided for participants to complete each record (eg, 7-10 AM) to support their engagement in the daily diary procedure; variability in response timing will be modeled empirically, such as through adjustment for the interval between consecutive assessments in the computation of MSSD [24] or lagged associations in dynamical structural equation models [67]. To address overlap in the time period covered by morning and evening assessments, evening reports serve as the main measure to capture day-to-day change in affective experiences. Morning assessments were included to examine affective change over the course of each day (see “Statistical Analyses” section). For example, morning assessments facilitate examination of (1) persistence or change in positive and negative affect over the course of each day and (2) persistence or change in positive and negative affect from one day to the next morning. Finally, participants will complete scheduled longitudinal assessments online 6, 12, and 18 months after baseline assessment. Due to the schedule of participant recruitment, longitudinal follow-up assessments will end in May 2026.

### Measures

#### Baseline Assessment

Participants provided brief demographic information including age, biological sex, racial background, current grade level, each parents’ level of education, number of people living in the household, and household income. Participants also reported on objective indicators of, and subjective perception of, socioeconomic status. Specifically, participants completed the Family Affluence Scale [88] on which they reported (1) the number of computers at home; (2) whether their family owns a car, van, or truck; (3) whether they have their own bedroom; (4) the number of family holidays taken last year; (5) whether their family has a dishwasher; and (6) the number of bathrooms in their home. Prior studies demonstrate the test-retest reliability, convergent validity, construct validity, internal consistency, and external validity of the Family Affluence Scale in adolescent samples [88]. Similarly, participants reported their perceived socioeconomic status relative to society at large and relative to their school on a ladder from 1 (low) to 10 (high) via the MacArthur Scale of Subjective Social Status—Youth Version [89]. Prior research in adolescent samples demonstrated internal consistency, cross-informant correlation, and test-retest stability of this measure over 2-6 months [89,90].

#### Academic Motivation

During baseline assessment, participants completed the high school version of the 28-item Academic Motivation Scale (Echelle de Motivation en Education) [91] to assess participants’ intrinsic and extrinsic motivation to engage with school including amotivation. Specifically, participants rated their agreement with each item on a 7-point Likert scale ranging from 1 (*does not correspond at all*) to 7 (*corresponds exactly*). Prior research on adolescent samples demonstrates construct validity, internal consistency, test-retest reliability, and discriminant validity of this measure [92].

#### Social Well-Being

Social well-being is indexed by adolescent report of perceived loneliness, social support, and relationship satisfaction. Specifically, participants indicated the severity of perceived loneliness through endorsement of 20 items on the UCLA Loneliness Scale version 3 [93] using a 4-point Likert rating scale from 1 (*never*) to 4 (*always*). Prior research demonstrates high internal reliability, construct validity, and convergent validity of the full and shortened measure to assess loneliness in adolescent populations [94].

Participants reported on the perceived availability of, and satisfaction with, social support via the 6-item version of the Social Support Questionnaire (SSQ) [95]. Specifically, for each of 6 situations, participants were asked to list all the people who may provide support and then to rate their satisfaction with the support available from 1 (*very dissatisfied*) to 6 (*very satisfied*). Previous research demonstrated the strong internal reliability, test-retest reliability, and construct validity of both the full and 6-item short versions of the SSQ in adolescent and undergraduate samples [95,96].

Finally, participants rated 75 items about their interpersonal relationships to assess overall attachment, trust, communication, and alienation in their relationship with their mother (or a mother figure), father (or a father figure), and peers on the Inventory of Parent and Peer Attachment (IPPA) [97]. Specifically, participants rated the accuracy of each item from 1 (*almost never or never true*) to 5 (*almost always or always true*). Prior research demonstrates strong internal consistency, test-retest reliability, and convergent validity of the IPPA to assess relationship quality in adolescent samples [97,98].

#### Mental Health

During baseline assessment, participants reported on their mental health as indexed by life satisfaction, perceived stress, difficulties in emotion regulation, broad psychosocial difficulties, conduct problems, hyperactivity- or inattention-related problems, peer relationship–related problems, prosocial behavior, and psychopathology including irritability, anxiety, depressive, and eating disorder syndromes. Additionally, a subset of participants completed the MINI-KID [99] to provide a categorical assessment of potential psychopathology.

Specifically, adolescents reported on life satisfaction by indicating agreement from 1 (*strongly disagree*) to 7 (*strongly agree*) with 5 statements about their overall quality of life or life satisfaction on the Satisfaction with Life Scale [100]. Prior research demonstrates reliability (ie, interitem reliability and internal consistency reliability) and validity (ie, construct validity, convergent validity, and criterion validity) of this measure to assess life satisfaction in adolescent samples [101].

Participants also indicated the severity of, and perceived ability to manage, stress on the 10-item version of the Perceived Stress Scale [102], which asked participants to rate the frequency with which they have been upset or had difficulty managing stress over the past month on 10 items rated from 0 (*never*) to 4 (*very often*). Prior research documented the convergent validity, internal consistency, criterion validity, and construct validity of the Perceived Stress Scale in adolescent samples [103].

Similarly, participants completed the Difficulties in Emotion Regulation Scale [104], which asked participants to rate the frequency with which they experience difficulties related to distressing emotions on 36 items from 1 (*almost always*) to 5 (*almost never*). Prior research demonstrates the interitem reliability and construct validity of the Difficulties in Emotion Regulation Scale in adolescent samples [105].

Adolescents completed the Strengths and Difficulties Questionnaire (SDQ) [106] to broadly assess psychosocial difficulties, conduct problems, hyperactivity- or inattention-related problems, peer relationship–related problems, prosocial behavior, and emotional distress over the past 6 months. Each participant was specifically asked to rate 25 items as *not true*, *somewhat true*, or *certainly true* and asked 5 questions to assess the impact of any difficulties reported (eg, the degree to which reported difficulties interfere at home or in the classroom). Prior research documented the interitem reliability, test-retest reliability, and construct validity of the SDQ to assess broad and specific domains of psychosocial difficulties in adolescent samples [107,108].

Participants completed the 13-item short version of the Mood and Feelings Questionnaire [109] to assess the severity of depressive symptoms over the past 2 weeks. Specifically, adolescents rated whether they experienced 13 depressive symptoms as *not true* (0), *sometimes* (1), or *true* (2). Prior research on the short version of the Mood and Feelings Questionnaire documented interitem reliability, internal consistency, test-retest reliability, criterion validity, convergent validity, and discriminative validity in adolescent samples [110].

Participants completed the 41-item Screen for Child Anxiety Related Emotional Disorders [111] to assess the severity of school avoidance and 4 anxiety syndromes common in childhood and adolescence: generalized anxiety, social anxiety or phobia, panic or somatization, and separation anxiety. Specifically, adolescents rated their agreement with 41 statements (eg, the ease with which they experience panic or somatization symptoms) from 0 (*not true or hardly ever true*) to 2 (*very true or often true*). The measure was modified slightly to assess the severity of anxiety symptoms over the past 2 weeks to align with participants’ concurrent assessment of depressive symptoms and capture change over time as suggested by Albano and colleagues [112] and Caporino and colleagues [113]. Prior research demonstrated strong psychometric properties of this measure to assess the severity of these 5 anxiety syndromes in adolescent samples including interitem reliability, test-retest reliability, concurrent validity, and discriminative validity [114] including over the past 2 weeks [112,113].

Participants also reported on the severity of irritability symptoms they experience via the Affective Reactivity Index (ARI) [115], which asks participants to rate agreement with 6 items that describe the ease with which they become angry or stay angry from 0 (Not True) to 2 (Certainly True). Prior research demonstrated strong psychometric properties of the ARI to assess irritability in adolescent samples including interitem reliability and convergent validity [115,116].

Participants reported on broad eating disorder symptoms (eg, body dissatisfaction and disordered eating behavior) via endorsement of 26 statements on the Eating Attitudes Test (EAT) [117] from 0 (*never*) to 3 (*always*), reported the frequency with which they engaged in 4 disordered eating behaviors (eg, binge eating) from *never* to *once a day or more*, and reported whether or not they lost 20 lb or more in the past 6 months. Prior research documents the interitem reliability, test-retest reliability, and convergent validity of the EAT-26 in adolescent samples [118].

The MINI-KID [99] was administered to assess categorical risk for various psychopathological conditions (eg, major depressive disorder, generalized anxiety disorder, and bulimia nervosa) excluding the following conditions due to relatively low prevalence in a community sample of adolescents [119-121]: tic disorders, psychotic disorders, and autism spectrum disorders. Prior research demonstrated the test-retest reliability, convergent validity, and discriminant validity of the MINI-KID in adolescent samples [99,122]. The MINI-KID was administered to a subset of 54 participants before it was discontinued due to time constraints for the full baseline assessment. Analyses involving the MINI-KID will be limited to participants who completed the interview.

#### Daily Diary Assessment

Twice daily for 35 days, participants completed brief surveys of positive and negative affect, depressive and anxiety symptoms, and stress on an iOS or Android OS device through the RealLife Exp app (LifeData LLC). Specifically, participants reported on positive and negative affect via completion of the Positive and Negative Affect Schedule—Child Form [123], on which they rated their experience of 30 emotions (eg, excited) since waking that morning from 1 (*not much or not at all*) to 5 (*a lot*). Prior research documented interitem reliability, internal consistency, convergent validity, and divergent validity of the Positive and Negative Affect Schedule—Child Form in adolescent samples [124-127].

Participants also reported twice daily on stressful experiences encountered since the last assessment including the context (ie, whether the stress is related to school, health, an interpersonal relationship, or general stress, such as the weather), severity, and duration of stress on 7 items adapted for adolescents from items previously developed by Dunkley and colleagues [128]. Specifically, adolescents’ experience of daily stress will be derived from 3 items that asked participants to reflect on the most bothersome event or issue experienced since the last assessment and then rate how unpleasant the event was from 1 (*not at all*) to 11 (*exceptionally*), how long distress lasted following the event from 1 (*a very brief amount of time*) to 7 (*a very large amount of time*), and how stressful the event was from 1 (*not at all)* to 11 (*exceptionally*). While Mandel and colleagues demonstrated internal consistency and validity of the measure in young adults [129], this study will be the first to examine the psychometric properties of this assessment of stress in the daily lives of adolescents.

Finally, participants rated depressive and anxiety symptoms twice daily via completion of symptoms drawn from the Dynamic Assessment of Depressive and Anxiety Symptoms [LM Rappaport et al, unpublished data, April 2025]. Specifically, regarding depressive symptoms, participants rated “hopelessness,” “worthlessness,” “sadness or depressed mood,” “feeling down on yourself,” “a feeling that things will not improve,” and anhedonia (“less interest in things you used to enjoy”) on an 11-point scale from 0 (*not at all*) to 10 (*severely*). Similarly, regarding anxiety symptoms, participants rated “nervousness,” “restlessness,” “difficulty relaxing,” “worry,” “trouble calming worry,” “difficulty breathing or shortness of breath (not due to exercise),” “trembling or shaking,” “heart pounding or racing,” “dizziness,” “tingling or numbness,” and “feeling jumpy” on an 11-point scale from 0 (*not at all*) to 10 (*severely*). This study will provide the first psychometric data to assess naturalistic variation in anxiety and depressive symptoms in adolescents’ daily lives.

### Follow-Up Assessments

Follow-up assessments include demographic information pertaining to age and grade. At each follow-up assessment, participants will report on academic motivation via the high school version of the Academic Motivation Scale [91]. Participants will also report on social well-being, specifically loneliness via the UCLA Loneliness Scale [93]; social support via the 6-item version of the SSQ [95]; and the quality of interpersonal relationships with a father figure, mother figure, and peers via the IPPA [97]. Finally, participants will report on mental health, specifically broad life satisfaction via the Satisfaction With Life Scale [100], irritability via the ARI [115], anxiety syndromes via the Screen for Child Anxiety Related Disorders [111], depressive symptom severity via the Short Mood and Feelings Questionnaire [109], eating disorder–related symptoms via the EAT [117], and broad psychosocial difficulties via the SDQ [106].

### Statistical Analyses

Prior to analysis, study data will be examined for outliers and missing data, the mechanism and correlates of which will be examined. From daily diary data, mean intensity of positive and negative affect will be computed for each participant as the mean of all the assessments of positive and negative affect provided. Variability and instability of positive and negative affect will be separately computed as the within-person standard deviation over time and MSSD between consecutive assessments, respectively [26,130]. Inertia in positive and negative affect will be computed by regressing positive and negative affect reported at each nightly assessment on positive and negative affect reported at the previous nightly assessment, respectively [65]. Finally, reactivity of positive and negative affect to stress will be computed via 2 approaches, which will be compared in subsequent data analyses as a sensitivity analysis. Initially, positive or negative affect reported at each nightly assessment will be regressed on stress reported concurrently while adjusting for positive or negative affect reported at the previous nightly assessment, respectively. Second, positive or negative affect reported at each nightly assessment will be regressed on stress reported concurrently while adjusting for positive or negative affect reported at the corresponding morning assessment of the same day, respectively. These 2 approaches effectively examine the degree to which participants’ report of stress experienced during the day is associated with change in positive or negative affect relative to affect reported the preceding day or the corresponding morning. Where possible, statistical analyses will use full information maximum likelihood estimation [131], which is robust to common forms of missing data [132]. Additionally, potential loss at follow-up will be addressed by identifying and then adjusting for predictors of attrition to mitigate potential bias in estimates of change in psychosocial well-being (eg, loneliness). To promote transparency and reproducibility and limit potential type II errors, empirical reports will follow statistical recommendations to present unadjusted *P* values [133,134]. Interpretation of empirical results will then adjust for multiple testing of correlated outcomes via the Benjamini-Hochberg correction [135].

Analysis of data from baseline assessment and daily diary assessment will use correlation and regression models, multilevel modeling, structural equation modeling, multilevel structural equation modeling, and DSEM to examine how emotion dynamics (eg, instability and inertia) correlate contemporaneously with each domain of well-being: psychopathology, academic motivation, and social well-being. For example, full and partial correlations will examine the association of baseline psychopathology (eg, irritability severity) with mean intensity of positive and negative affect and variability (ie, intraindividual standard deviation) or instability (ie, MSSD) of positive and negative affect over time while accounting for mean affective intensity, respectively. Regression and structural equation models will examine the association of mean intensity of, variability in, and instability in positive and negative affect with indices of academic motivation (eg, amotivation) and social well-being (eg, loneliness) while adjusting for concurrent psychopathology (eg, irritability severity). Model fit will be evaluated for each structural equation model [136] and multilevel structural equation model [137,138] to ensure that each model adequately represents the underlying data.

**Figure 2:**
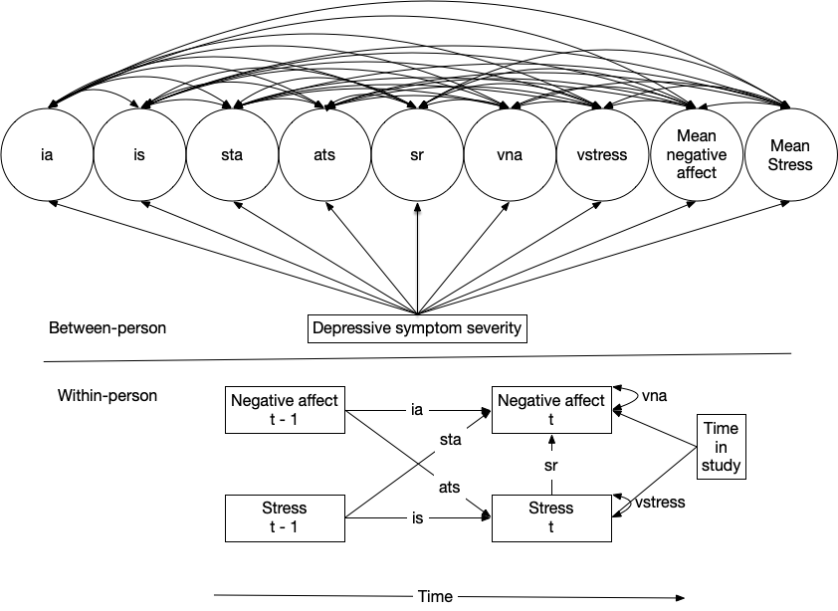
Example residual dynamic structural equation model of the contemporaneous association of psychopathology with emotion dynamics. “Time in study” represents the amount of time spent in the daily diary procedure at each record to adjust for potentially unique responding early in a daily diary procedure. ats: stress regressed onto prior affect; ia: inertia in affect; is: inertia in stress; sr: affective reactivity to stress; sta: affect regressed onto prior stress; vna: intraindividual variance in negative (or positive) affect; vstress: intraindividual variance in stress.

Similarly, dynamic structural equation models will examine how between-person variation in affective inertia in negative or positive affect and reactivity of negative or positive affect to stress correlate with adolescent psychopathology (eg, anxiety and depressive symptomatology) at baseline (Figure 2). Dynamic structural equation models can similarly examine the association of between-person variation in affective reactivity to daily stress with academic motivation and social well-being above and beyond concurrent psychopathology, mean overall intensity of affect (eg, negative affect) and stress, and intraindividual variability in affect and stress. Specifically, inclusion of psychopathology (eg, depressive symptom severity) assessed at baseline will facilitate examination of how between-person variation in affective inertia and affective reactivity to stress correlate contemporaneously with psychopathology and whether between-person variation in affective inertia and affective reactivity to stress are associated with academic motivation or social indices of well-being above and beyond psychopathology [139,140]. As previously suggested [24,141], analyses of emotion dynamics (eg, variability and affective inertia) will adjust for between-person variation in mean positive and negative affect.

Similarly, analyses of data from longitudinal follow-up assessments will use multilevel modeling, structural equation modeling, multilevel structural equation modeling, and DSEM to examine how each emotion dynamic (eg, instability and inertia) assessed at baseline may predict change in each well-being domain. Specifically, change in each domain will be examined through 2 approaches: estimation of latent growth curves that model trajectories of change (eg, in peer relationship quality) over the 18 months of planned follow-up assessments and estimation of latent change score models that examine change (eg, in academic amotivation) between consecutive follow-up assessments [142]. Similar to analyses of concurrent data from baseline assessment, analyses of emotion dynamics will adjust for mean positive and negative affect [24].

Within the latent growth curve approach, terms that describe the trajectory of change in each well-being domain (eg, intrinsic academic motivation) will be regressed on each emotion dynamic (eg, inertia in positive affect) assessed at baseline to evaluate the degree to which each emotion dynamic may predict gradual change in each well-being domain over the 18-month follow-up period. Subsequent analyses will adjust for psychopathology (eg, symptoms of anxiety syndromes) assessed at baseline and at each follow-up assessment to evaluate the degree to which each emotion dynamic may predict change in each well-being domain above and beyond concurrent, traditional assessment of psychopathology.

Within the latent change score approach, structural equation models and the between-person level of both multilevel and dynamic structural equation models will model change in each well-being domain (eg, social well-being) between consecutive follow-up assessments. Broadly, this model captures within-person change over each 6-month period, which will be, in turn, regressed on each emotion dynamic assessed at baseline to evaluate whether each emotion dynamic (eg, instability in positive affect) may predict change in each well-being domain over the subsequent 6, 12, or 18 months. Finally, secondary analyses will use latent change score models to examine the multivariate patterns of change among psychosocial constructs assessed at baseline and in follow-up assessments (eg, psychopathology and social well-being outcomes). For example, planned analyses will examine whether qualities of adolescents’ relationships with parents and peers (eg, trust, communication, and alienation) prospectively predict or follow from preceding changes in psychopathology over time.

## Results

This study was funded in July 2020, but the project was delayed, and funding extended, for 2.5 years due to research restrictions required during the COVID-19 pandemic. Between April 2023 and November 2024, a total of 152 adolescents were enrolled into this longitudinal study such that all scheduled 6-month follow-up assessments were completed by May 2025, 12-month follow-up assessments were completed by November 2025, and 18-month follow-up assessments will be completed by May 2026 (see Figure 1 for a timeline of the study). Three participants (3/152, 1.97%) withdrew, which leaves an effective sample size of 149. Regarding statistical power, the effective sample size reflects 74.5% (149/200) of the target sample, which provides 80% statistical power to detect similarly moderate correlations at baseline using a cautious testwise a value of .005 (*r*=0.290) and 80% statistical power to detect a moderate prospective association (β=.380) of each emotion dynamic with change in each well-being domain, even after accounting for 20% attrition.

## Discussion

### Principal Findings

This study aims to examine adolescents’ socioemotional development by examining emotion dynamics that describe fluctuations in naturalistic positive and negative affect. Specifically, this study aims to clarify the implications of putative emotion dynamics (ie, mean affective intensity, variability, instability, inertia, and affective reactivity to stress) for psychosocial well-being both concurrently and prospectively over 18 months. For example, by examining the association of emotion dynamics with well-being outcomes (eg, social well-being and academic motivation), this study aims to identify how specific affective patterns (eg, reactivity of negative affect to stress) contribute to adolescent psychosocial adjustment including change in adolescent well-being over time. Finally, this study operationalizes psychosocial well-being broadly to include myriad indicators of psychopathology (eg, symptoms of depressive, eating, and anxiety syndromes) as well as life satisfaction, academic motivation, and social relationships. Traditional approaches to studying adolescent mental health and well-being primarily relied on static, trait-level assessments that may not fully capture the complexity of affective fluctuations in adolescents’ day-to-day lives [45]. Therefore, this study seeks to identify the potential contribution of assessing state variation in affect (eg, intraindividual variability in positive and negative affect) to predict psychosocial development during adolescence. For example, this study seeks to identify the potential incremental contribution of assessing state variation in affect over traditional static assessment (eg, baseline self-report assessments) and mean affective intensity.

If study findings align with hypotheses, it will emphasize the contribution of assessing dynamic variation in affect beyond traditional, static assessment, such as traditional self-report assessments of depressive and anxiety symptoms [22]. For example, we hypothesize that naturalistic emotion dynamics derived from in situ affective fluctuations may inform the concurrent model of, and prospective prediction of change in, adolescents’ psychosocial well-being. Moreover, this study will clarify whether naturalistic emotion dynamics inform the prospective prediction of change in adolescent psychosocial well-being (eg, depressive symptomatology) above and beyond traditional, static assessments at baseline, such as baseline self-reports of depressive symptomatology. Insights into the importance of assessing dynamic variation could emphasize the importance of understanding and assessing adolescents’ daily experiences of positive and negative affect for research and clinical assessment. Such insights may also highlight the potential of emotion dynamics as markers of developmental risk and resilience in adolescence. For example, results from this study may clarify the contribution of dynamic affective variation to adolescent psychosocial development and facilitate more accurate identification of risk for psychopathology including not only *who* is at risk but *when* one enters a period of acute risk (eg, for a depressive episode or panic attack) to support timely and tailored intervention.

This study will also evaluate the degree to which putative affective fluctuations inform change in adolescent well-being beyond clinical symptomatology. Prior research predominantly examined putative emotion dynamics as correlates of psychopathology or broad life satisfaction (see Dejonckheere et al [24] and Houben et al [26] for review). Through examination of academic motivation, social well-being, and life satisfaction, this study will examine the implications of emotion dynamics for functional outcomes previously approximated by clinical symptomatology. If empirical study findings align with hypotheses, this study will highlight the broad relevance of in situ affective fluctuations for adolescent psychosocial development beyond the specific clinical outcomes often studied. Evidence that emotion dynamics contribute to the prediction of change in adolescent psychosocial well-being above and beyond psychopathology would suggest the clinical relevance of emotion dynamics to predict prognosis and functional outcomes above and beyond the existing, traditional assessment of psychopathology. Such evidence would also demonstrate the relevance of emotion dynamics for education, counseling, and social work (eg, through the identification of students at risk for social isolation or decreased academic motivation). Overall, this study uses a broad assessment of adolescent psychopathology and psychosocial well-being in a longitudinal design to assess the breadth and potential relevance of emotion dynamics for adolescent psychosocial development.

Conversely, if study findings do not align with hypotheses, this may suggest that systematic intraindividual variation in emotion adds minimal predictive value for long-term psychosocial outcomes beyond traditional static measures [24]. Although emotion fluctuates over time, weak associations may clarify which dynamics most influence adolescent well-being. Through the use of Bayesian methods to estimate associations, this study will evaluate the relative contributions of emotion dynamics to change in psychopathology and psychosocial well-being over and above concurrent, baseline psychopathology. Evidence of weak associations of emotion dynamics with change in psychopathology or psychosocial well-being may highlight the relevance of emotion dynamics for short-term outcomes, such as daily functioning, over chronic, long-term psychosocial or clinical outcomes in adolescence. Given the substantial socioemotional developmental challenges posed by adolescence [2], the present examination may clarify which emotion dynamics influence adolescents’ mental health, academic well-being, and social functioning contemporaneously or prospectively over time.

### Limitations

This study is one of the first and one of the largest longitudinal studies to date to investigate the naturalistic manifestation of emotion dynamics in adolescence as well as their implications for change in adolescent psychosocial well-being over time. However, it is not without limitations. The present sample size represents an initial exploration into the manifestation and potential implications of emotion dynamics. However, this study is designed to inform future research with larger samples to provide greater clarity on the magnitude of each empirical association. As such, empirical findings may be interpreted as a foundation to guide future research.

Similarly, the generalizability of study findings may be limited by sample characteristics, including cultural, socioeconomic, and geographic factors. Participants were drawn from the Windsor-Essex region; factors such as household income, urban versus rural residence, parental education, and access to community resources could influence daily affective patterns and their association with psychosocial outcomes. However, this study is designed to inform future research with larger samples, which could leverage a multisite design to evaluate and increase the generalizability of study empirical findings.

Additionally, this study focuses on daily stressors (eg, daily hassles) rather than traumatic or major life stressors (eg, medical illness, financial hardships, deaths, and victimization). Daily stressors provide a meaningful index of stress in investigations of well-being [143,144] and may predict psychopathology and psychosocial well-being above and beyond major life events [145,146]. However, empirical results regarding associations of daily stressors with well-being should not be generalized to trauma or major stressful events.

Moreover, when designing daily diary and related protocols, one must consider the burden on participants who complete daily diary surveys multiple times daily for many consecutive days (see Shiffman et al [46] for review). Therefore, daily diary surveys are generally restricted in length or frequency, particularly in studies of children and adolescents [147]. This study used a twice daily survey procedure to ask a relatively large set of questions (eg, about affect, stress, and internalizing symptoms) while minimizing the participant burden that could result from many daily assessments. Although this study sampled adolescent participants less often than some extant experience sampling methodology studies of affect in adolescent samples [58,148], daily assessments are consistent with other assessments of affect and stress in adolescence [149,150] and adulthood [151,152]. Therefore, consistent with evidence of affective variation at multiple timescales [153,154], this study is primarily designed for evening assessments to inform interday variation in positive and negative affect while morning assessments provide some information on intraday change in positive and negative affect. We also note that naturalistic assessments of adolescents may often occur less frequently than assessments of adults or young adults (undergraduate students [155]). However, future research is needed to clarify the manifestation and implications of rapid, intraday variation in positive and negative affect to adolescent well-being. Similarly, the 35-day daily diary period may fail to assess longer-term cycles [151], which could be examined in future research on change in emotion dynamics over time [153].

Finally, this study lacks measures of contextual factors that may impact affective fluctuations or individuals’ responses to stressors (eg, social support provided in situ). For example, daily and momentary assessments of sleep quality [156], diet [157], and exercise [158] have been associated with well-being. Instead of measuring contextual factors, this study prioritized the assessment of between-person variability in psychosocial factors (eg, social support and self-efficacy) that may be associated with within-person processes (eg, reactivity of positive and negative affect to stress). Therefore, this study was designed to prioritize the examination of between-person correlates of dynamic affective processes. However, future research is needed to develop comprehensive biopsychosocial models of in situ variation in positive and negative affect, such as by integrating active or passive assessments of contextual factors (eg, sleep and exercise).

### Conclusions

This protocol paper outlines a longitudinal study designed to examine the developmental implications of naturalistic emotion dynamics (ie, affective intensity, variability, instability, inertia, and affective reactivity to stress). Specifically, this study examined how each dynamic correlates with and prospectively predicts the development of adolescent psychosocial well-being over time including and beyond psychopathology. This paper provides a comprehensive record and overview of the study’s aims, background, methodology, and procedures to promote transparency and reproducibility. Additionally, this paper details the integration of daily diary assessment within a longitudinal study design to examine the developmental implications of naturalistic, in situ affective fluctuations. Thereby, beyond serving as a methodological reference, this protocol paper also establishes a framework for future research on adolescent psychosocial and socioemotional development by demonstrating how daily diary assessment can advance the study of dynamic affective processes beyond traditional, static assessment. Particularly following needed replication of any empirical findings, this study has the potential to clarify the manifestation of daily affective fluctuations in adolescence as well as their implications for broad adolescent well-being to, ultimately, inform research and clinical practice in psychology, counseling, and education.

## Abbreviations

ARI: Affective Reactivity Index
DSEM: dynamic structural equation modeling
EAT: Eating Attitudes Test
IPPA: Inventory of Parent and Peer Attachment
MINI-KID: Mini International Neuropsychiatric Interview for Children and Adolescents
MSSD: mean squared successive difference
SDQ: Strengths and Difficulties Questionnaire
SSQ: Social Support Questionnaire
